# Supplementary material for: A high-resolution mRNA expression time course of embryonic development in zebrafish
Source: eLife. 2017 Nov 16;6:e30860. doi: 10.7554/eLife.30860 (PMC5690287; doi:10.7554/eLife.30860)
Supplement: Supplementary file 6. [file elife-30860-supp6.zip › biolayout-clusters-files/Cluster095-genes.html]

Cluster095


# Cluster095: Genes

| Ensembl ID | Gene Name | Chr | Start | End | Biotype |
| --- | --- | --- | --- | --- | --- |
| ENSDARG00000100791 | TRIL | 19 | 19916637 | 19918913 | protein\_coding |
| ENSDARG00000018881 | apobec2a | 22 | 603619 | 613996 | protein\_coding |
| ENSDARG00000077938 | cd248b | 21 | 26456999 | 26459041 | protein\_coding |
| ENSDARG00000034933 | chchd3b | 25 | 3232957 | 3243584 | protein\_coding |
| ENSDARG00000087198 | cthrc1a | 19 | 12995970 | 13007561 | protein\_coding |
| ENSDARG00000077826 | efcc1 | 11 | 26381517 | 26429011 | protein\_coding |
| ENSDARG00000029019 | epb41b | 16 | 26576173 | 26614727 | protein\_coding |
| ENSDARG00000103092 | grem2b | 12 | 47545974 | 47560418 | protein\_coding |
| ENSDARG00000004455 | p2rx5 | 5 | 41497453 | 41520774 | protein\_coding |
| ENSDARG00000097728 | si:dkey-23a23.3.1 | 4 | 29185114 | 29187996 | lincRNA |
| ENSDARG00000103586 | si:dkey-65j6.2 | 23 | 43308307 | 43359601 | protein\_coding |
| ENSDARG00000094753 | si:dkey-71d15.2 | 21 | 34895108 | 34909440 | protein\_coding |
| ENSDARG00000054878 | six2b | 12 | 25518659 | 25520810 | protein\_coding |
| ENSDARG00000021948 | tnc | 5 | 27960853 | 28025364 | protein\_coding |
